# The Book World of Medicine and Science

**Published:** 1893-09-23

**Authors:** 


### THE BOOK WORLD OF MEDICINE AND SCIENCE.

### SEPTEMBER REVIEWS.

THE *Nineteenth Century* opens with a paper on the physical causes of weariness, being the substance of the Rede Lecture lately delivered at Cambridge by Professor Michael Foster. The effect of fatigue on the nervous system is illustrated by various interesting experiments, and the article should certainly be studied attentively by that large class of persons who cultivate the theory that weariness is a species of original sin to be trampled out by strong measures. A curious anecdote may be quoted showing the effect upon muscular movement of mental exertion. "An Italian professor determined, by means of the apparatus which we have been using to-day, the amount of work which he could on a certain morning do before he was stopped by weariness. He then set himself to two hours' hard mental work, and the form of work he chose was that of examining candidates for their degree. I believe there exists a theory among the junior members of the University that while being examined is very hard work, examining is a light and airy task. I would ask them to re-examine that theory by the light of the following fact: The professor, so soon as the two hours' examination was over, went back to his apparatus and found that his power of bending his finger was enormously cut down. I understand that a candidate was used as a sort of control experiment. It was found he could work the apparatus as well after as before the examination; it is added that he had not 'satisfied the examiners.'"

The *Contemporary Review* is greatly enlivened this month by one of Mr. Andrew Lang's excursions into the region of "Comparative Psychical Research." The object of the article is to show that the "motives, methods, and results" of the modern spiritualists, if not as old as the hills, are at any rate closely paralleled by proceedings accurately reported so long ago as 1665. The leading personage in the earlier "Society for Psychical Research" was the famous Dr. Greatrakes, known as the "Irish Stroker." He was undeniably successful in his dealings with hypochondriacal and hysterical patients. "He used to chase the disease up and down their bodies if it did not fly out through the interstices of his fingers, and if he could drive it into an outlying part and then forth into the wide world, the patient recovered." But many more lively wonders were witnessed by the seventeenth century body of investigators, including the sight of a gentleman's butler floating in the air, and the ghost of a dun in the person of an elderly woman who came after a matter of twenty-eight shillings which was owing to her, and vanished to the sound of "most delicate music" when the debt was discharged. The minor phenomena connected with the manifestations are curiously similar in all recorded instances, and may well provoke Mr. Lang's final query, "Is there such a thing as persistent identity of hallucination among the sane?"

Mr. Frank Spence, in an article on "How to Stop River Pollution," urges that the same system of inspection which has been found to work well in purifying the atmosphere under the Alkali Works Regulation Act, should be applied to liquid discharges so that the effluents of all sewage works should be subject to tests. He contends "the only measures needful will be to insist that every outflow of waste liquid from sewage works should pass through an open conduit,

**THE BOOK WORLD OF MEDICINE AND SCIENCE**—*continued.*

accessible to the inspector at all hours of the day and night." The local authorities to be allowed to erect any kind of works and adopt any purifying process they pleased, but be held rigidly responsible for the result.

In the *Fortnightly Review* Mr. W. Bevan Lewis has a very able investigation into "The Origin of Crime." His conclusions are not altogether cheerful, since he shows that while inebriety is still on the increase, "alcoholism tends towards the production of epilepsy, and the epileptoid state in the offspring, and when indulged in to excess by this degenerate progeny tends to issue in the convulsive forms of insanity so often associated with criminal propensities." There is nothing very new in his demonstration of the close connection of drunkenness with both insanity and crime, but it is a connection which cannot under the existing state of the legislation be pointed out too clearly or too often.

Mrs. Percy Frankland has some interesting pages in *Longman* on Bacterial Life and Light. Air and sunlight have always of late years been favourite weapons in the war against disease, but it is only quite recently that science has detected the bactericide action of the sun, and been able to show exactly why it is beneficial. Mrs. Frankland quotes a German physician of a hundred years ago for the dictum, "Our houses, hospitals, and infirmaries will without doubt some day be like hot-houses, so arranged that the light, even that of the moon and stars, is permitted to penetrate without let or hindrance." Whether the moon and the stars will ever recover their prestige in matters medical, is doubtful. Their new rival, electric light, is far inferior still to the sun, for it has been shown that whereas two or three hours of sunlight are sufficient to destroy the typhoid bacillus, six hours' exposure to 1,000 candle power electric lamp was required to produce on it the same effect. The effect of sunlight in the purification of water is very remarkable. "Some boiled water contained in a flask was inoculated with an immense number of a bacillus, closely resembling the typhoid organism, normally present in the body, and frequently found in water, the "bacillus coli communis." So many were introduced that nearly 100,000 individuals were present in every 20 drops of the water. This flask, then, containing water so densely sown with microbes, was placed in the sunshine for one hour, whilst another and similar flask was kept during the same time in the dark. On being subsequently examined it was ascertained that whereas a slight increase in the number of bacilli had taken place in the dark flask, in the insolated flask absolutely no living organisms whatever were present."

*The National Review* has a timely notice on Hops and Hop-pickers by Mr. Charles Edwardes. Until the last few years hop-picking afforded the only opportunity available to the slums for getting a glimpse of the country, and in spite of many hardships and some rough doings the annual outing works on the whole for good. "The perfume of the hop-field is one of its pleasantest characteristics. It is a bitter sweet like nothing else. I heard an old woman picker remark, as she sniffed it eagerly, 'I feels better soon as ever I has it in my nose.'" This dame swore by it as a remedy for rheumatism. It is also popularly supposed to be a purifier of the blood, I believe it is a fact that at no time has the cholera got a footing in the hop districts of England. As a soporific the scent is admittedly valuable." Mr. Hodgson contributes an earnest defence of sport under the title of "The Immorality of Evolutionary Ethics." The contention is that no methods of slaughtering animals for food presents so little cruelty as those practised by the sportsman.

